# Findings from Community Workshops Designed to Help Expand Arizona's Dementia Capable System

**DOI:** 10.1093/geroni/igab046.2760

**Published:** 2021-12-17

**Authors:** David Coon, Aylin Angulo, Marielysse Cortes, Berta Carbajal, Kassey Stotler, zenya Weatherall, jami Goldman

**Affiliations:** Arizona State University, Phoenix, Arizona, United States

## Abstract

Among the 5.8 million people living with Alzheimer’s disease (AD), there are three vulnerable groups where community partners can join efforts to serve the community more comprehensively. These include (a) people living alone with Alzheimer’s disease and other related dementias (ADRD) who may or may not have a family caregiver, (b) people with Down Syndrome or another intellectual or developmental disability aging with ADRD and their family caregivers, and (c) people with ADRD and their family caregivers in the Latino community. Dementia capable systems are designed to address the needs and concerns of all individuals, families, and communities impacted by ADRD. The project develops and expands ADRD programs and services across Arizona through educational workshops, case management services, and evidence-based programs. Workshops (N=67) were provided to a variety of professionals and community members ranging from promotores/CHW’s (community health workers) and case managers to family caregivers and people living with dementia (N=2,272). Workshops successfully attracted a substantial proportion of Hispanic or Latino/a participants (63%) and women (84.5%). Perception of benefit ratings were overwhelmingly positive with over 90% of participants agreeing or strongly agreeing that: the workshops met expectations; they were willing to attend other programs by us; and they learned something they could use. Moreover, based on their workshop experience, they felt more confident that they could help these three underserved populations. Overall, workshops were clearly acceptable to participants and feasible to deliver. In addition, they contributed to an increased awareness in ADRD related to the project’s three target groups.

